# Predictors of Life Satisfaction Among Older Adults in Long-Term Care Settings

**DOI:** 10.1093/geroni/igaf122.2694

**Published:** 2025-12-31

**Authors:** Xiaoli Li, Cheng Yin, Elias Mpofu

**Affiliations:** Southern Illinois University, Carbondale, Illinois, United States; University of North Texas, Denton, Texas, United States; University of North Texas, Denton, Texas, United States

## Abstract

Resident satisfaction is a critical indicator of the quality of care in long-term care facilities. Yet, the relative importance quality of care factors in predicting resident satisfaction remains unclear for guiding resident support initiatives. This study aims to identify the predictors of satisfaction LTCF residents. This cross-sectional study enrolled a sample of 399 older adult residents from LTCFs in Shanghai, Nanjing, and Changsha, China. Hierarchical multiple regressions were utilized to examine self-report survey data on ten resident personal variables, and seven care facility service domains were examined for their relative contribution to resident care satisfaction. Moreover, the analysis included the interaction between personal factors and care service attributes.

**Results:**

The combined personal and care service factors explained 26.1% of the variance in overall resident satisfaction. Personal factors that predicted resident satisfaction included age, level of independence, and length of stay (ΔR2 = 0.11). Of the care facility factors, the significant predictors of higher resident satisfaction were spending time (β = 0.60, p < 0.01, ΔR2 = 0.09) and environment domains (β = 0.62, p < 0.01, ΔR2 = 0.03). Age moderated the relationship between the spending time domain and overall satisfaction, with a positive effect for residents aged 70–79 compared to those aged 60–69 (β = −1.26, p < 0.05).

**Conclusions:**

This study provides evidence to suggest the importance of personal and care facility characteristics to LTFC resident satisfaction. Based on the findings, improved resident satisfaction is likely with LTCF care services that provide tailored care plans using resident characteristics.

